# An Evaluation of Health-Related Quality of Life in Children with Nasal Septum Deviation

**DOI:** 10.3390/children9111714

**Published:** 2022-11-09

**Authors:** Lechosław Paweł Chmielik, Grażyna Mielnik-Niedzielska, Anna Kasprzyk, Tomasz Stankiewicz, Artur Niedzielski

**Affiliations:** 1 Department of Pediatric Otolaryngology, Centre of Postgraduate Medical Education, 01-809 Warsaw, Poland; 2 Department of Pediatric ENT, Children’s Hospital in Dziekanów Leśny, 05-092 Dziekanów Leśny, Poland; 3 Department of Pediatric Otolaryngology, Medical University of Lublin, 20-093 Lublin, Poland; 4 Independent Otoneurological Laboratory, Medical University of Lublin, 20-093 Lublin, Poland

**Keywords:** nasal cavity, quality of life, rhinoplasty

## Abstract

Background: From the 1950s, the quality of life criterion came to be studied in earnest, originally forming a part of measurement of human development in Western Europe and the USA. The present study aims to compare the health-related quality of life (HRQL) between children with nasal septum deviation and healthy children controls. Materials and Methods: Subjects were children suffering from nasal septum deviation, one of the commonest chronic diseases of the upper respiratory tract. Controls were randomly recruited from kindergarten, primary and secondary schools (junior high school & high school). All schools and subjects were randomly selected. The CHQ-PF50 questionnaire was used and outcome scores were calculated by an algorithm for the 13 tested HRQL variables. Results: Means for all outcome scores in the test subjects (i.e., children with deviated nasal septums) varied between 3.65–89.27 with a standard deviation between 0.83–25.66 and a median between 3.4–100 (*n* = 101). Those for the controls (*n* = 102) were 3.78–97.11, 0.86–14.21 and 4.40–100, respectively. Test subjects showed significant scoring declines in Physical Fitness, Role/Social–Emotional/Behavioral, Role/Social–Physical, Mental Health, Self-esteem, General Health Perceptions, Parental Impact Emotional and Time and Family Limitations in Activities. Conclusions: 1. The well-being of children with nasal septum deviation was found to be chiefly limited by their physical fitness, effects of physical condition on social behavior/interaction and how health is perceived. 2. Parents considered their children’s health to be paramount, as demonstrated by assessing the HRQL.

## 1. Introduction

Nasal deformations to bone and cartilage within the septum and bone pyramid may lead to chronic obstruction of the nasal cavities, which thereby reduces the operating efficiency of the upper respiratory tract [1,2,3]. Such changes are found in 15% to 39% of children visiting their GP (General Practitioner) when complaining of impaired breathing through the nose [4,5]. A study by Gray has shown that curvature of the nasal septum never straightens spontaneously when children are progressively growing, excepting the neonatal period [6]. Children’s nasal deformities can have various etiologies. The most common include post-traumatic changes occurring during the peri, intra and postnatal periods. Genetics or disproportionate facial development are also of some importance [7]. Developing a classification of nasal septal deviations has now allowed improved communication and evaluation between hospital centers and patients’ doctors. One of the most frequently used breakdowns into divisions of nasal septum curvature is the one according to Cottle [8] and Mladina [9]. Disruptions to the breathing pattern can affect the physical and mental development of a child. However, it now possible to effectively use septo and rhinoplasty during child development thanks to the method of reconstructive nasal septum plasticity, as introduced by Cottle, which leaves the growth dynamics of the nose undisturbed [10]. Paranasal sinuses and ears become infected very often if the nasal patency deteriorates. 

Quality issues in contacts between parents and children may arise, along with parents’ free time becoming limited, whenever inflammatory changes occur for extended periods, as well as if there are problems in how daily family life is organized (e.g., reconciling working hours with taking offspring to ENT clinic appointments and in providing childcare during times of any sickness). The questionnaire selected was one found appropriate to this study. When large populations are studied, general-purpose questionnaires will usually suffice for assorted pathologies, allowing comparisons to be made between healthy controls and test disease subjects, irrespective of the actual number of study participant subjects. However, such questionnaires are not suited for measuring discrete changes for individuals. New HRQL questionnaires have thereby been prepared that are specific to the case being researched, which can sensitively detect any changes during defined periods. These have been used to investigate the effectiveness of any given treatment or in monitoring lesion progression. They are, however, unsuitable for patients suffering from concomitant diseases. The present study has aimed to compare HRQL between children with nasal septum deviation and healthy children controls.

## 2. Materials and Methods

The study subjects tested were children treated as hospital inpatients for clinical symptoms of nasal septum deviation—one of the most common chronic diseases of the upper respiratory tract. Those eligible were aged between 5 and 18 years, were not suffering from any acute disease and had questionnaires that had been correctly completed. Exclusion criteria were those with ages outside the 5–18 years range, those suffering from acute disease and/or other chronic disease and those with questionnaires that had been incorrectly filled in. Controls were recruited from kindergarten, primary and secondary schools (junior high school & high school) in Warsaw and its environs. All schools and subjects were randomly selected. A module in the STATISTICA software package enabled statistical power to be determined in order to establish appropriate sample sizes in all the study groups. 

A general-purpose questionnaire was used in this study: the Child Health Questionnaire—Parent Form 50 CHQ-PF-50 (CHQ-PF50). It is based on psychometric tests that evaluate the physical and mental health/well-being of children aged 5–18 years. It was first introduced by JM Landgraff and JE Ware in 1994 [11] and has since found extensive use in comparing HRQL between sick and healthy children. Any given health-status profile can be thereby evaluated, including aspects of physical/mental health such as dealing with emotions, behavior and in making social contacts. It consists of 50 questions posed to parents or legal guardians which are divided into 13 groupings, where the evaluation period depends on the content of these groupings; however, there was no specific time limit for those grouping concerned with how health and family cohesion were perceived in general. A comparison was made between present health status and that from one year prior. For all other questions, the preceding four weeks only were investigated. Responses to all questions were assigned an appropriate numerical value. An algorithm was used to calculate results, consisting of: summed values obtained divided by numbers of posed questions, out of which the smallest possible value is deducted. The answer so calculated is then further divided by the range of outcomes possible to give final scores ranging from 0 to 100, where the greater the score is, the more desirable the well-being and life functioning [12]. The Polish version of the general-purpose CHQ-PF50 survey was used in this study. This has been validated and available since 2001.

The study was conducted according to the guidelines of the Declaration of Helsinki, and approved by the Ethics Committee of the Komisja Bioetyczna przy ’Warszawskim Uniwersytecie Medycznym’ (approval code KBO/12/11, dated 15 March 2011).

The STATISTICA software page was used to perform all statistics. A *p* ≤ 0.05 value was taken as being significant and two-tailed tests were chosen at the researchers’ discretion. Significant p values are shown in red. 

Variables are all defined in the questionnaire and are divided into discrete and continuous, where the former are subdivided into those having 2-point distributions or n-point distributions, for which the indices of structure and counts were computed. For the continuous variables, summary statistics were calculated, comprising counts, arithmetic mean, standard deviation, minimum/maximum, skewness and kurtosis as well as positional statistics of median, Q25 and Q75. The majority of continuous variables were non-normally distributed thus non-parametric statistics were used; nevertheless, in the interests of data mining, parametric testing was used at times. 

The non-parametric tests used were the Mann–Witney Median test (with associated rank correction), the Kruskall–Wallis test—for performing multiple comparisons of mean ranks—and the median test. Whenever appropriate, the parametric test equivalents were used, i.e., t-tests and ANOVA. The F-test was used to check the equality of variance, whilst multiple comparisons employed the RIR-Tukey test. Correlation coefficients were calculated by the Tukey test and Spearman’s Rank test.

The χ^2^ test of independence was used in independence analyses for the discrete variables, whereas the 2-tailed exact test was conducted for 4-field tables if numbers turned out lower than expected. Whenever tables had more fields, appropriate groupings were adopted. Wanke’s surplus values were determined in the contingency tables to facilitate interpretation.

## 3. Results

A total of 150 parents of children with deviated nasal septums (test subjects) received CHQ-PF50 questionnaires, out of which 101 were eligible (67.33%). These children comprised 48 girls and 53 boys (average age 12 years, ranging from 5–18) (Table 1).

Children with nasal septum deviation exhibited mean values for all continuous variables ranging from 3.65–89.27, standard deviations ranging from 0.83–25.66, whilst the medians ranged from 3.40–100.00.

The quality of life for the test subjects was found to be limited in the least degree within the following areas: Physical Functioning (PF) at 89.27, Role/Social–Emotional/Behavioral (REB) at 88.78 and Role/Social–Physical (RP) at 86.8. In contrast, the greatest limitations were observed in: General Health Perceptions(GH) at 61.17, Parental Impact–Emotional (PE) at 64.93 and Family Cohesion (FC) at 65.15.

Eligible control subjects consisted of 50 girls and 52 boys, ranging from 5–18 years of age (mean 10.58, standard deviation 0.86–14.21 and median 3.78–97.11). The mean range of all the continuous variables in this group was 3.78–97.11 (Table 1), with standard deviations ranging from 0.86–14.21 and medians lying between 4.40–100. The smallest limitations in quality of life for the controls were found within the following areas: Physical Functioning (PF) at 97.11, Role/Social–Emotional/Behavioral (REB) at 96.51 and Role/Social–Physical (RP) at 96.24. 

The greatest limitations in quality of life for the controls were, however, found within the following areas: Family Cohesion (PC) at 66.57, General Health Perceptions (GH) at 75.41 and Parental Impact–Emotional (PE) at 77.21.

The control group was compared with children suffering from nasal septum deviation where significant differences between groups for each HRQL variable are highlighted in Table 2.

The median test demonstrated significant differences between the test group and healthy control group in the following areas of well-being: evaluating the general health condition of the child (STAND), discomfort and pain (BP), behavior (BE) and family cohesion (FC). Those areas showing statistical significance were found when well-being declined in the test group for the following areas: physical condition/fitness (PF), the impact of behavior or emotional state on functioning socially (REB), the effect of social functioning being limited by physical health (RP), how mental health is perceived (MH), self-esteem (SE), how general health is perceived (GH), the effect that children’s health has on parental emotions (PE), parental free time being limited by their children’s health (PT) and when joint family activities are limited (FA).

## 4. Discussion

At present, we are not aware of any studies as comprehensive as this having been performed on the quality of life for children suffering from upper respiratory tract disorders. One of the main problems are incomplete questionnaires on children suffering from chronic diseases when treated as outpatients. Return rates of fully completed questionnaires are reportedly around 70% [13,14], which closely agrees with the 67.33% rate observed in this study.

Most studies that describe quality of life in patients with nasal septum deviation are, however, on adults, assessing the outcomes of surgical treatment of nasal septum by using specific tests. Nevertheless, three studies have been found in the literature that assess the quality of life in children with nasal septal deviation [15,16,17], where most used specific tests to assess the effects of nasal septum surgery. Patients suffering from a deviated nasal septum have described having chronically obstructed nasal cavities that reduce the functional efficiency of the upper respiratory tract [15,16,17]. Disturbances in children’s breathing patterns may lead to aberrations in mental and physical development. In the latter case, whatever the cause, this can lead to deteriorating physical fitness, which is recognized to be, inter alia, a factor determining peer-group status and thus a child’s self-esteem may suffer as a consequence. 

The following symptoms are encountered when the quality of life significantly deteriorates: agitation/uneasiness, tiredness and a blocked-up nose [15,16,17].

We were, however, unable to find any studies that compared the quality of life in children with nasal septal deviation to healthy children using general-purpose tests.

This study has shown mean values in individuals’ quality of life areas ranging from 61.17 to 89.27. The worst assessed areas were, firstly, the perception of general health (GH) at 61.17, followed by the small effect that children’s health has on parental emotions (PE) at 64.93 and then family cohesion (FC) at 65.15. Physical fitness (PF) was, however, rated the highest at 89.27 and the impact of behavior or emotional state on functioning socially (REB) was closely behind at 88.78, along with social functioning being limited by physical health (RP) at 86.80. 

The control group and test group (children with nasal septum deviation) did not significantly differ in terms of deteriorating quality of life when assessing current health status. The quality of life was, however, found to significantly decline for the following: mobility (PF), the impact of behavior or emotional state on functioning socially (REB), the effect of social functioning being limited by physical health (RP), how mental health is perceived (MH), self-esteem (SE), how general health is perceived (GH), the effect that children’s health has on parental emotions (PE), parental free time being limited by their children’s health (PT) and when joint family activities are limited (FA).

## 5. Conclusions

1. The evaluation of HRQL in children suffering from diseases of the upper respiratory tract is a valuable means for determining general health status and for monitoring the treatment course.
2. The greatest limitation to well-being in children with curvature of the nasal septum is observed in physical fitness, the effect of social functioning being limited by physical health and how health is generally perceived.
3. Evaluating a healthy child’s HRQL shows that a given child’s health is of paramount importance to their parents.

## Figures and Tables

**Table 1:** Summary statistics—continuous variables for the test group (deviated nasal septum) and for the control group.

Control	N	N	Std. Dev	Std. Dev	Mean	Mean	Q25	Q25	Min.	Min.	Q75	Q75	Median	Median	Max	Max
variables for	1	2	1	2	1	2	1	2	1	**2**	1	2	1	2	1	2
STAND	101	102	0.83	0.86	3.65	3.78	3.40	3.40	1.00	1.00	4.40	4.40	3.40	4.40	5.00	5.00
PF	101	102	14.04	5.17	89.27	97.11	83.33	94.44	44.44	77.78	100.00	100.00	94.44	100.00	100.00	100.00
RP	101	102	21.51	9.92	86.80	96.24	83.33	100.00	16.67	50.00	100.00	100.00	100.00	100.00	100.00	100.00
REB	101	102	18.89	7.49	88.78	96.51	88.89	100.00	33.33	66.67	100.00	100.00	100.00	100.00	100.00	100.00
BE	101	102	18.09	11.15	74.56	79.19	64.17	71.67	30.00	55.00	89.17	89.17	76.67	80.83	100.00	100.00
BP	101	102	25.66	16.75	75.94	85.39	50.00	70.00	10.00	10.00	100.00	100.00	80.00	90.00	100.00	100.00
SE	101	102	15.48	14.07	75.45	80.19	66.67	70.83	29.17	37.50	87.50	91.67	79.17	83.33	100.00	100.00
MH	101	102	19.77	13.62	69.55	79.80	55.00	70.00	15.00	30.00	85.00	90.00	75.00	80.00	100.00	100.00
PE	101	102	22.62	14.21	64.93	77.21	50.00	66.67	8.33	41.67	83.33	91.67	66.67	75.00	100.00	100.00
GH	101	102	15.86	13.12	61.17	75.41	51.67	68.33	20.83	29.17	72.50	85.00	60.00	76.67	97.50	100.00
FA	101	102	21.32	12.90	74.71	85.29	21.32	75.00	20.83	50.00	58.33	95.83	79.17	89.59	100.00	100.00
PT	101	102	22.29	11.60	79.10	90.41	22.29	88.89	11.11	66.67	66.67	100.00	88.89	88.89	100.00	100.00
FC	101	102	22.87	18.66	65.15	66.57	22.87	60.00	0.00	0.00	60.00	85.00	60.00	60.00	100.00	100.00

The following is a list of abbreviations used in the table below: 1—continuous variables for the test group (deviated nasal septum), 2—continuous variables for the control group, SE—self esteem, REB—role/social–emotional/behavioral, BE—general behavior, MH—mental health, FC—family cohesion, FA—family limitations in activities, PF—physical functioning, RP—role/social–physical, STAND—assessment of the general condition of the child, GH—general health perceptions, BP—bodily pain/discomfort, PT—parental impact-time, PE—parental impact-emotional.

**Table 2:** A comparison of children suffering from deviated nasal septums (test group) with controls.

	Control Means	Test Means	Control Medians	Test Medians	P t. Medians
STAND	3.78	3.65	4.40	3.40	0.0567
REB	96,51	88,78	100.00	100,00	0.0265
PF	97.11	89.27	100.00	94.44	0.0003
BP	85.39	75.94	90.00	80.00	0.9354
BE	79.19	74.56	80.83	76.67	0.1653
RP	96.24	86.80	100.00	100.00	0.0003
SE	80.19	75.45	83.33	79.17	0.0060
MH	79.80	69.55	80.00	75.00	0.0029
PE	77.21	64.93	75.00	66.67	0.0364
GH	75.41	61.17	76.67	60.00	0.0000
FC	66.57	65.15	60.00	60.00	0.6265
FA	85.29	74.71	89.59	79.17	0.0136
PT	90.41	79.10	88.89	88.89	0.0263

The following is a list of abbreviations used in the table below: SE—self-esteem, REB—role/social–emotional/behavioral, BE—general behavior, MH—mental health, FC—family cohesion, FA—family limitations in activities, PF—physical functioning, RP—role/social–physical, STAND—assessment of the general condition of the child, GH—general health perceptions, BP—bodily pain/discomfort, PT—parental impact–time, PE—parental impact–emotional.

## Data Availability

All results are available from the corresponding author.

## Abbreviations

CHQ-PF-50	Child Health Questionnaire-Parent Form 50
PF	Physical Functioning
RP	Role/Social-Physical
GH	General Health Perceptions
BP	Bodily Pain/Discomfort
PT	Parental Impact-Time
PE	Parental Impact-Emotional
REB	Role/Social Emotional–Behavioral
SE	Self Esteem
MH	Mental Health
BE	General Behavior
FA	Family Limitations in Activities
FC	Family–Cohesion
STAND	Assessment of the general condition of the child
